# Supplementary material for: An empirical, 21st century evaluation of phrenology
Source: Cortex. 2018 Sep;106:26–35. doi: 10.1016/j.cortex.2018.04.011 (PMC6143440; doi:10.1016/j.cortex.2018.04.011)
Supplement: Multimedia component 1 [file mmc1.pdf]

Supplementary Materials

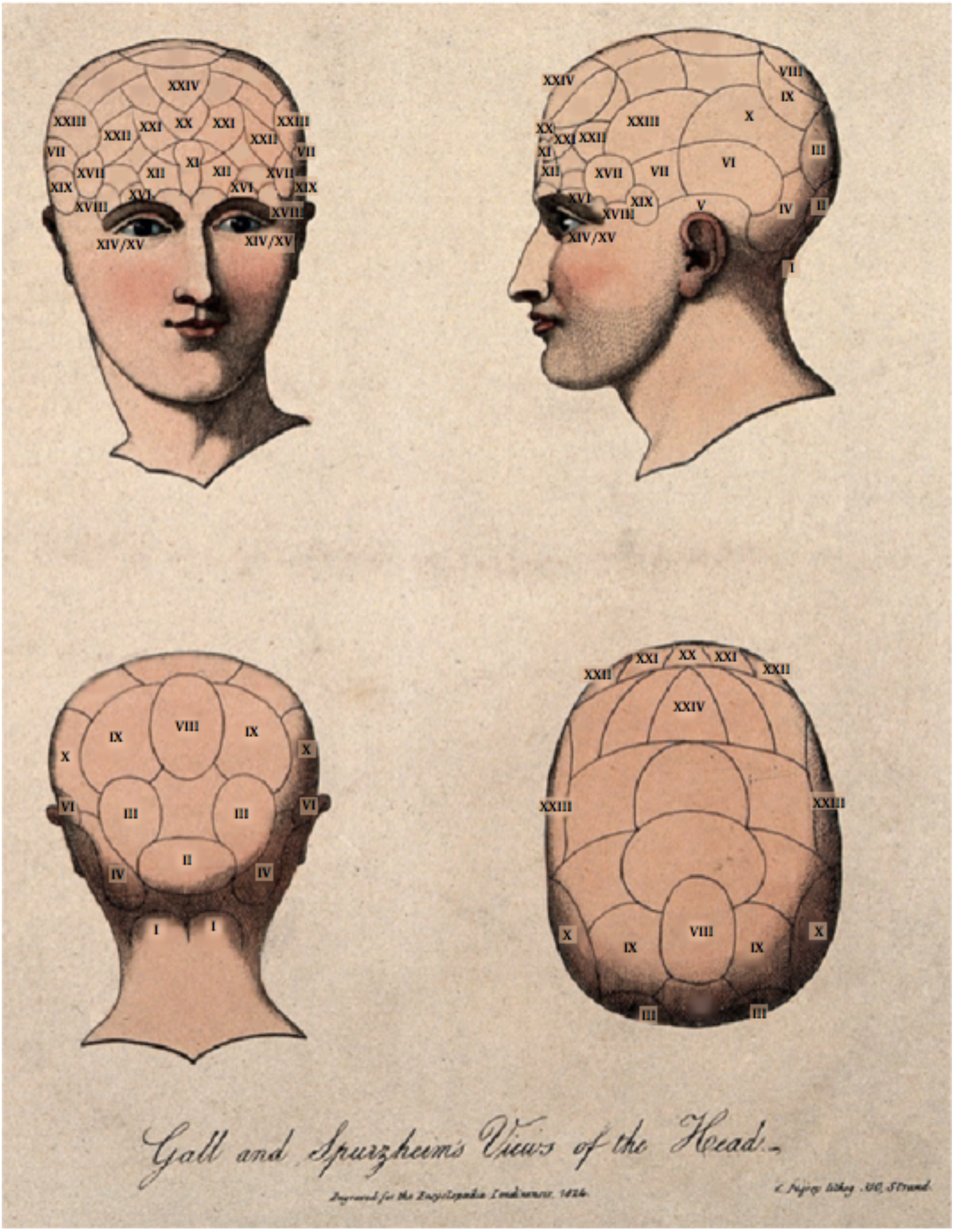

Supplementary Figure 1: Phrenological organs. The 23 roman numerals (I-XII, XIV-XXIV) correspond to the faculties explored in the present paper (Gall 1835). To account for the extra parcels, note that Gall's original 27 faculties were revised to 33 by Spurzheim (1815). Later phrenologists continued to revise both the number and organisation of phrenological organs. Adapted from "Physiognomy Plate I" (Wilkes 1825).

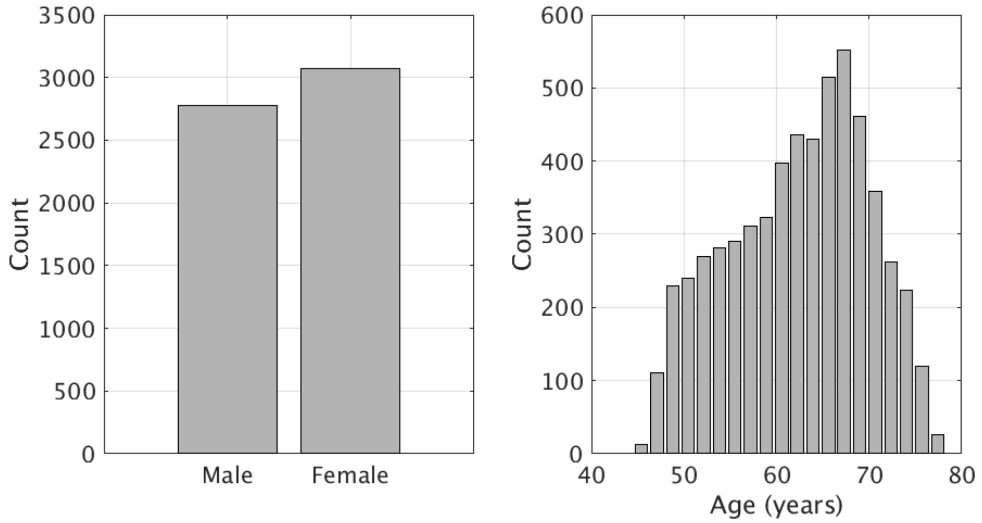

Supplementary Figure 2: Sex and age of subjects.

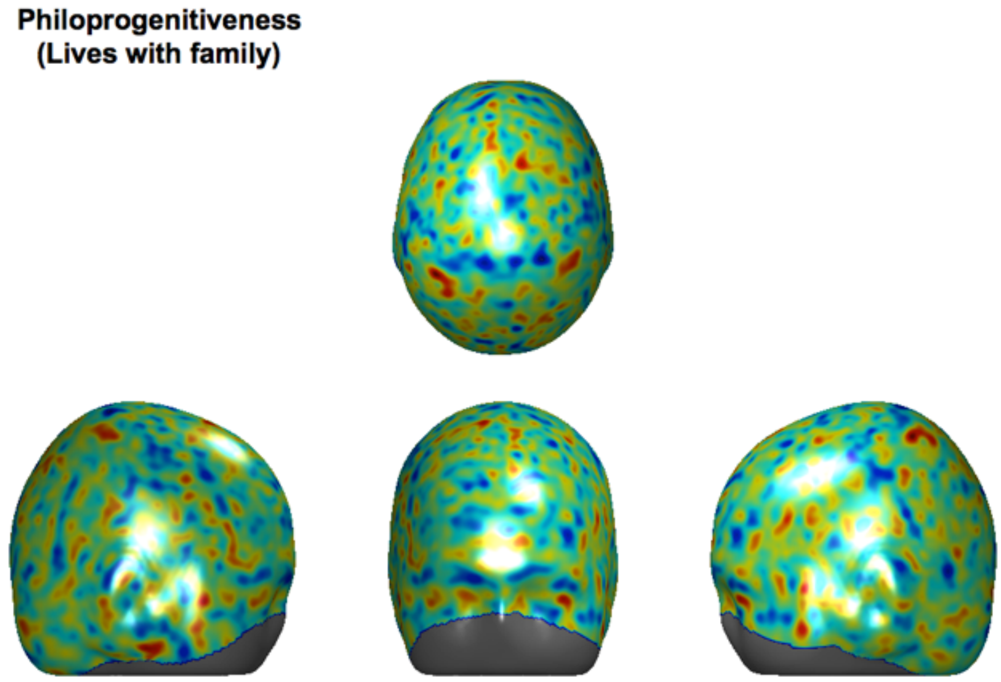

Supplementary Figure 3: Faculty II.

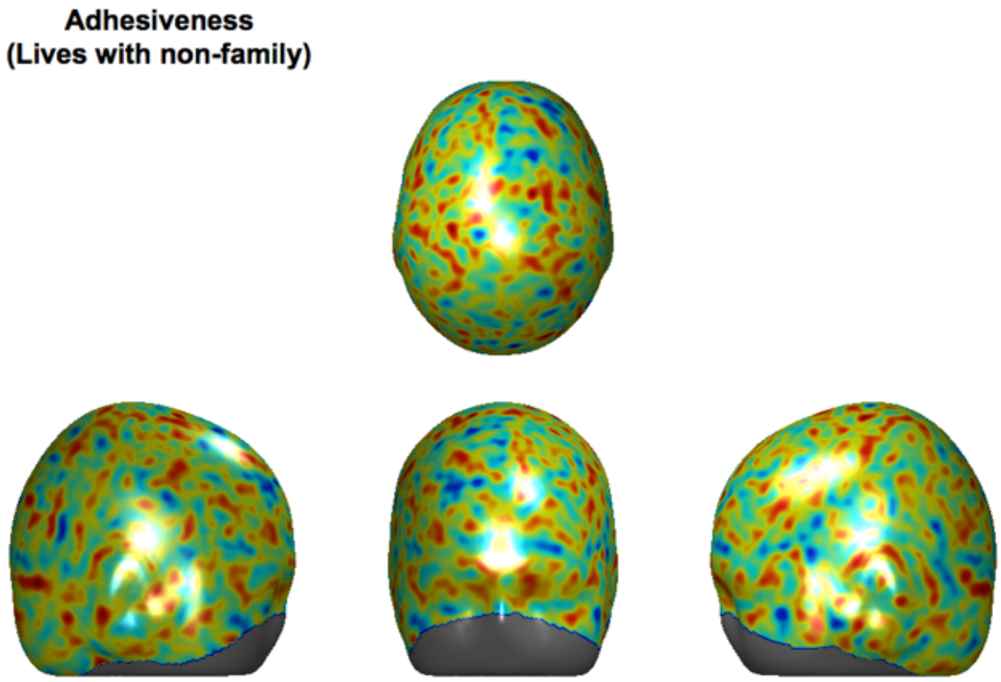

Supplementary Figure 4: Faculty III.

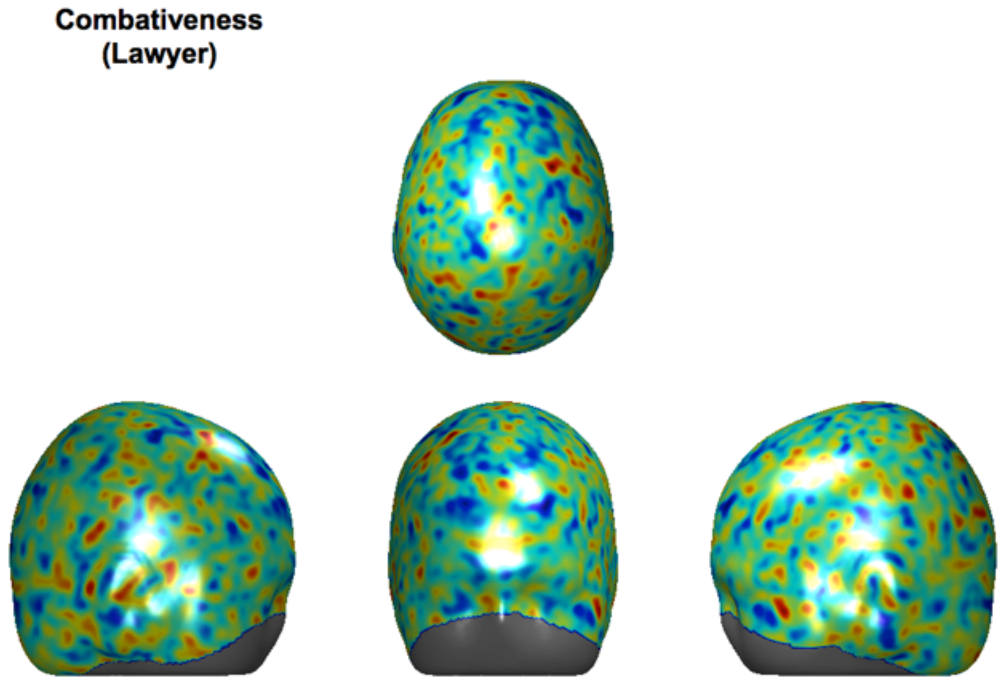

Supplementary Figure 5: Faculty IV.

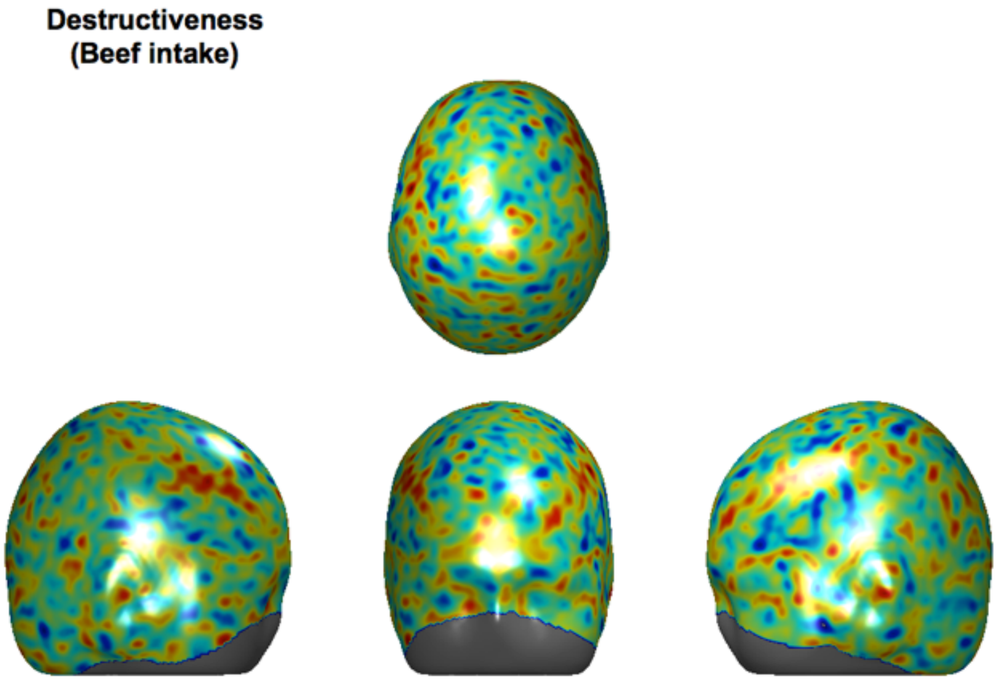

Supplementary Figure 6: Faculty V.

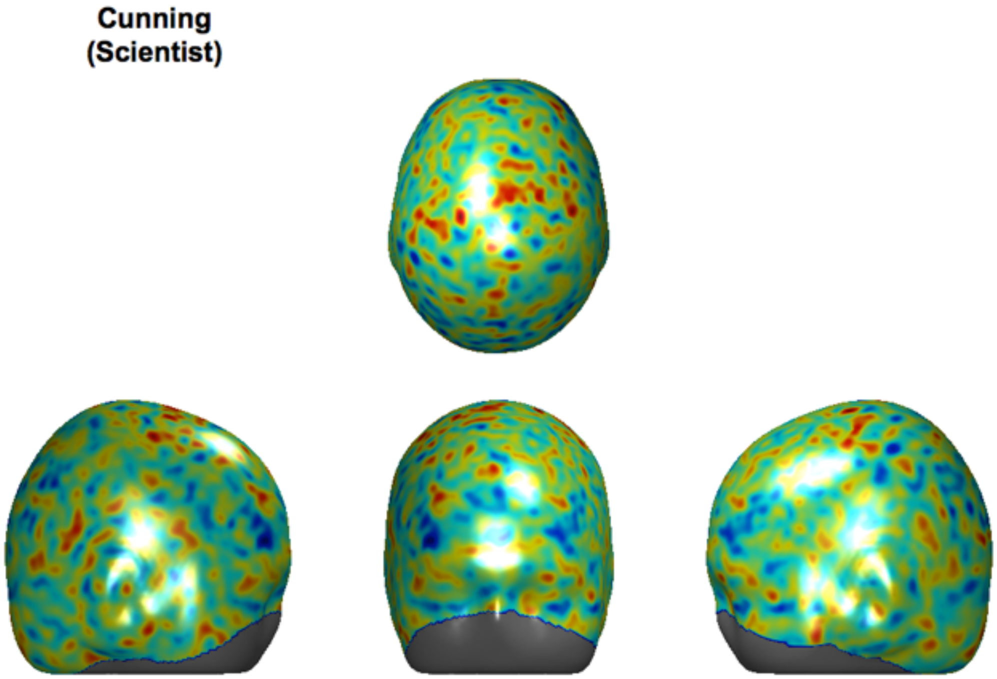

Supplementary Figure 7: Faculty VI.

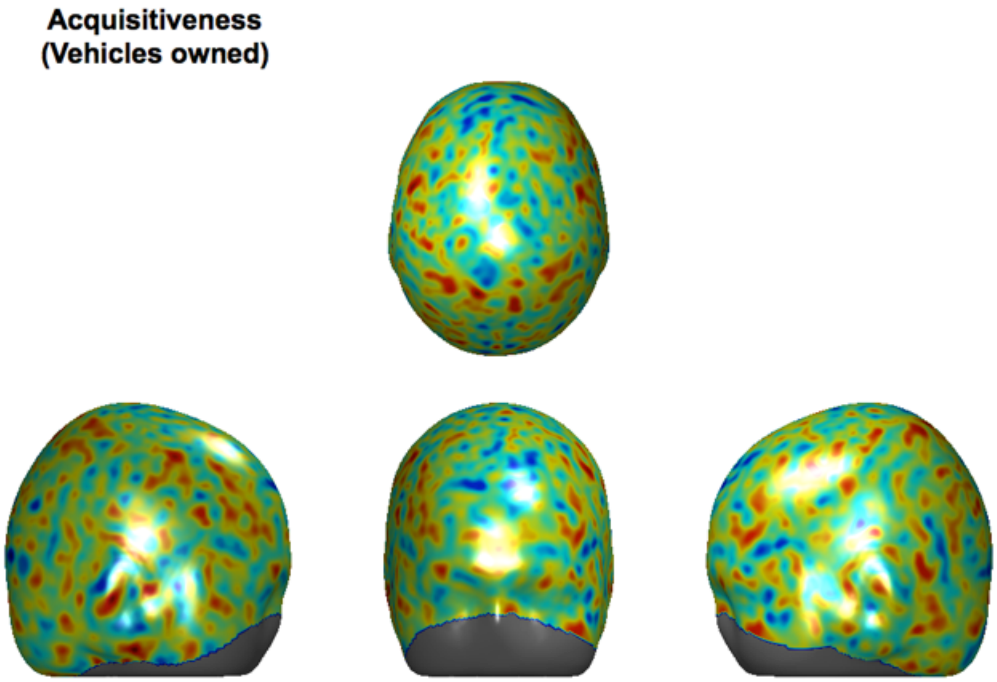

Supplementary Figure 8: Faculty VII.

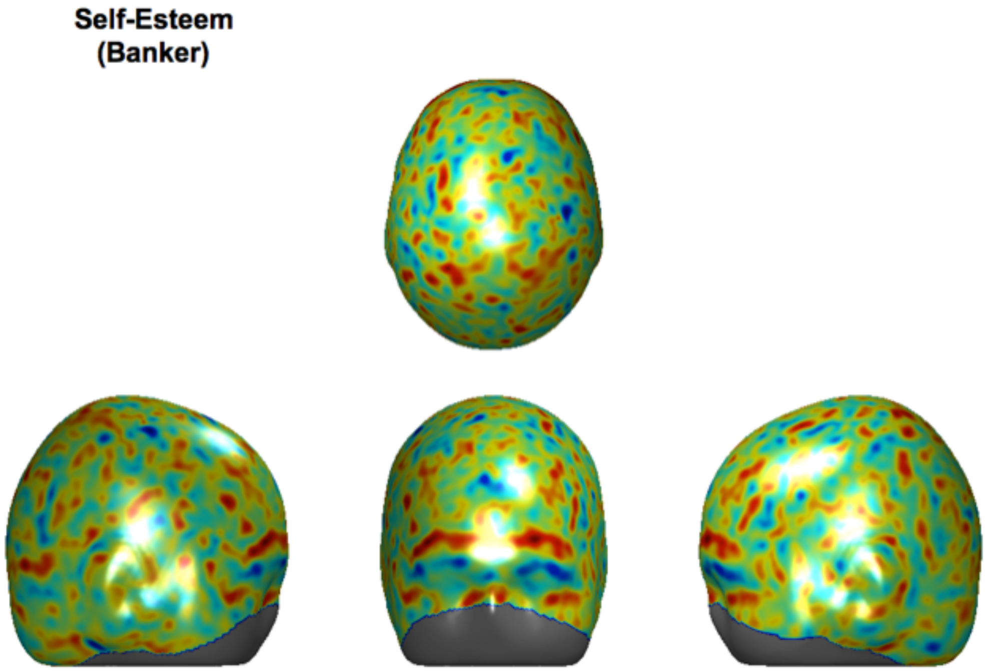

Supplementary Figure 9: Figure VIII.

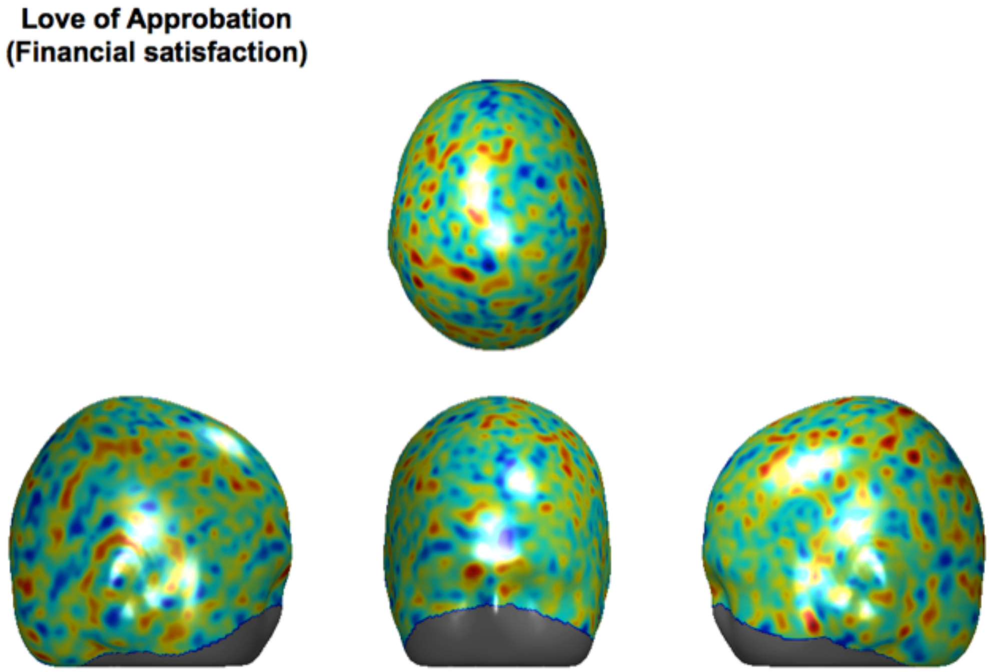

Supplementary Figure 10: Faculty IX.

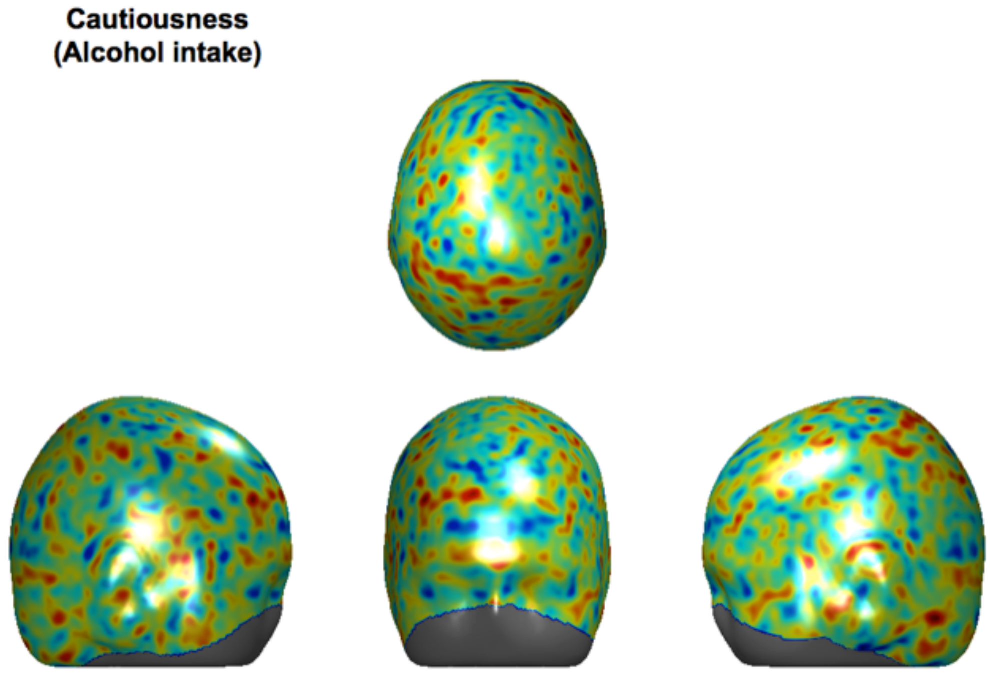

Supplementary Figure 11: Faculty X.

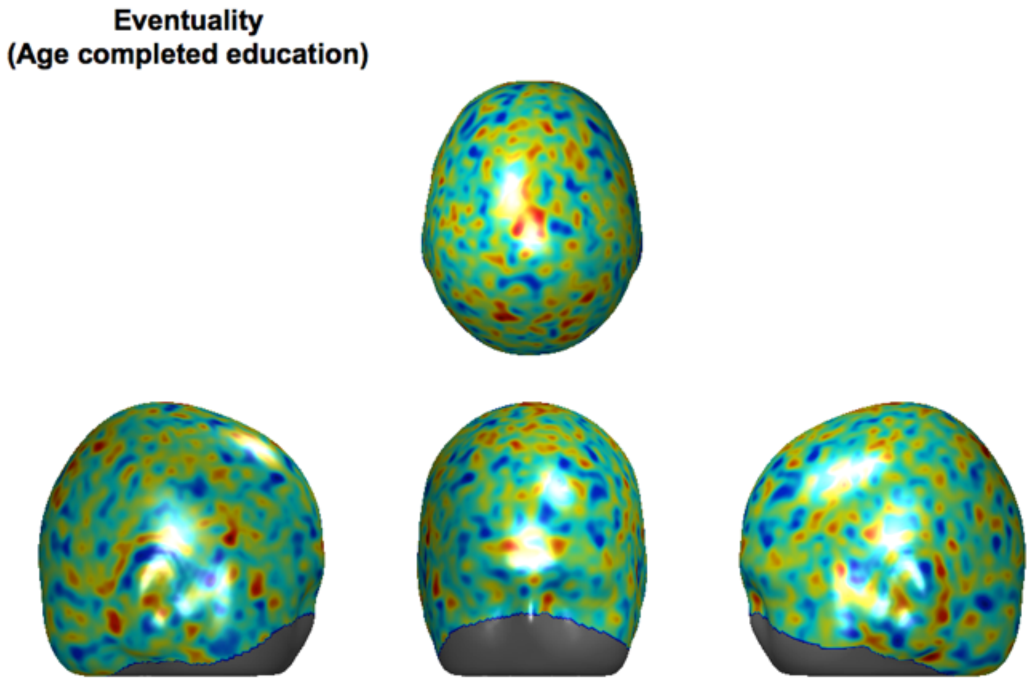

Supplementary Figure 12: Faculty XI.

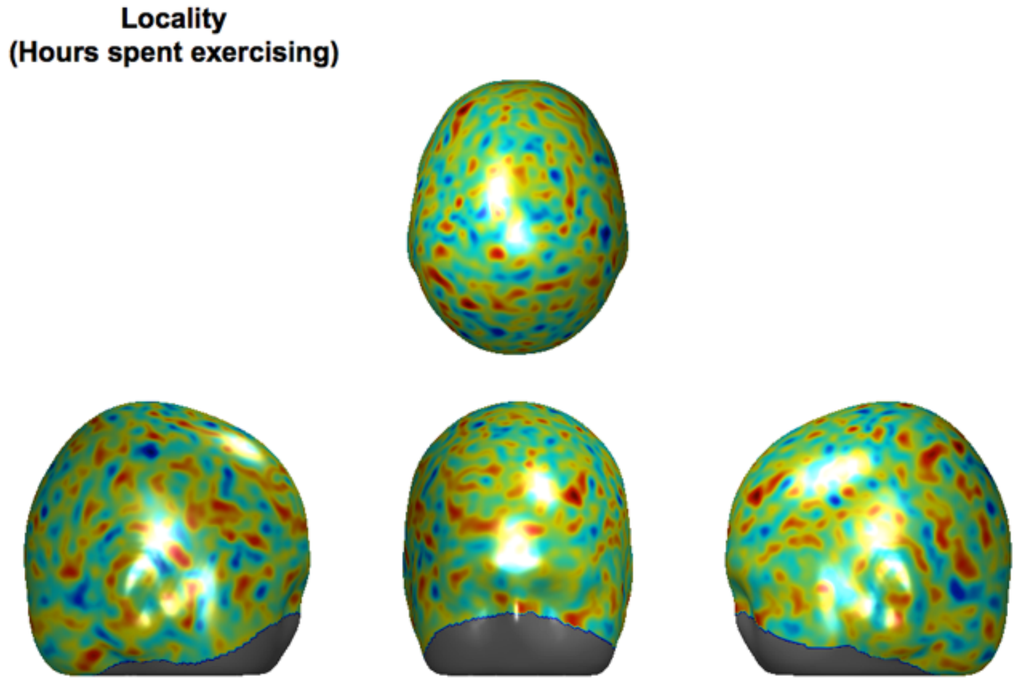

Supplementary Figure 13: Faculty XII.

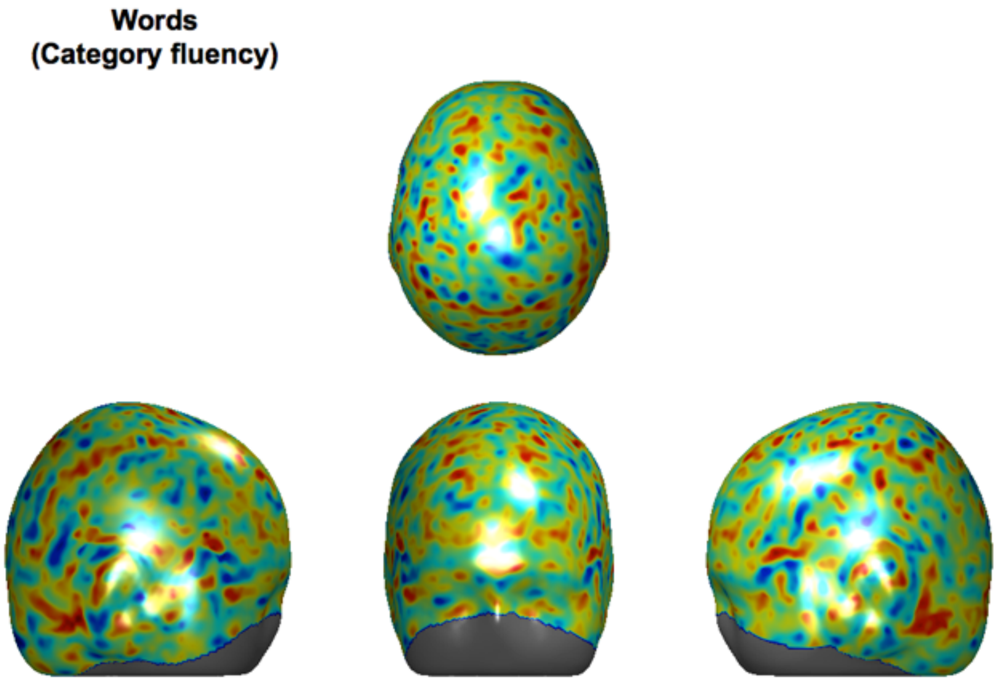

Supplementary Figure 14: Faculty XIV.

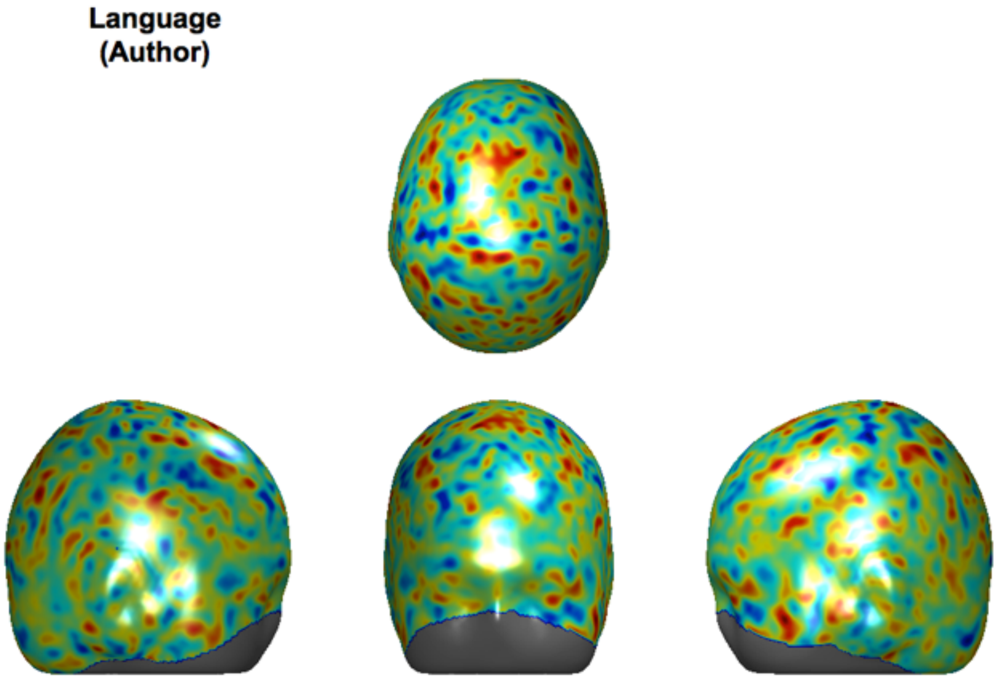

Supplementary Figure 15: Faculty XV.

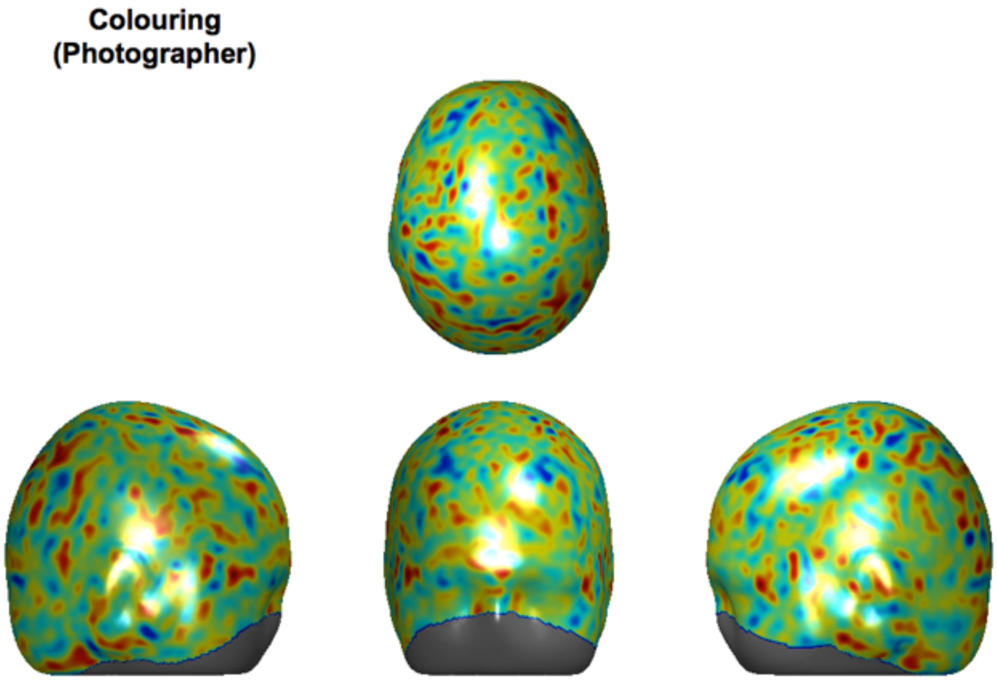

Supplementary Figure 16: Faculty XVI.

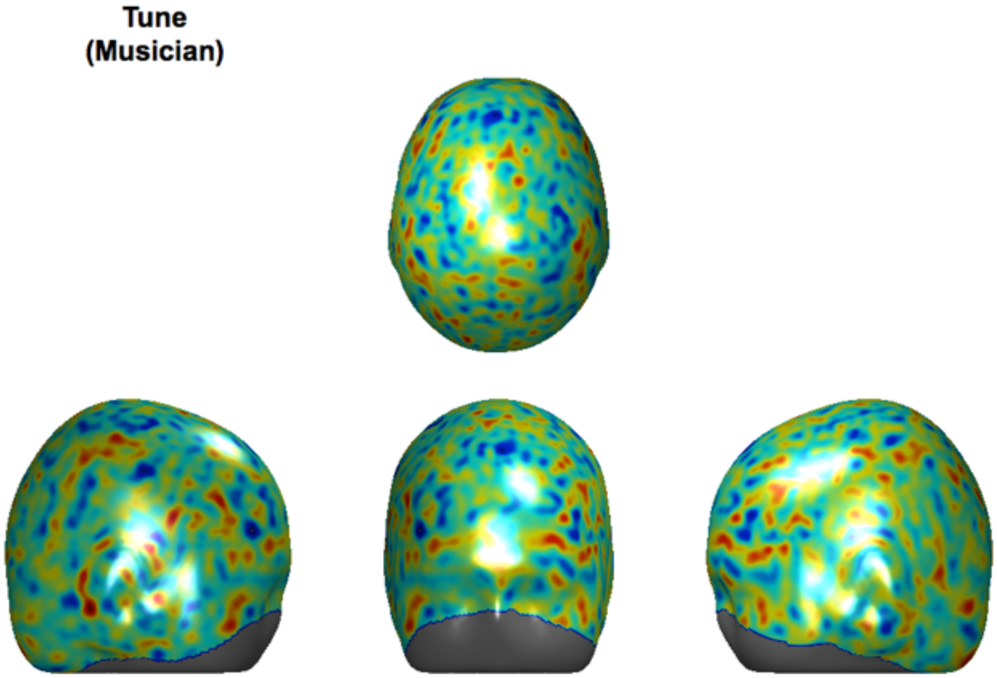

Supplementary Figure 17: Faculty XVII.

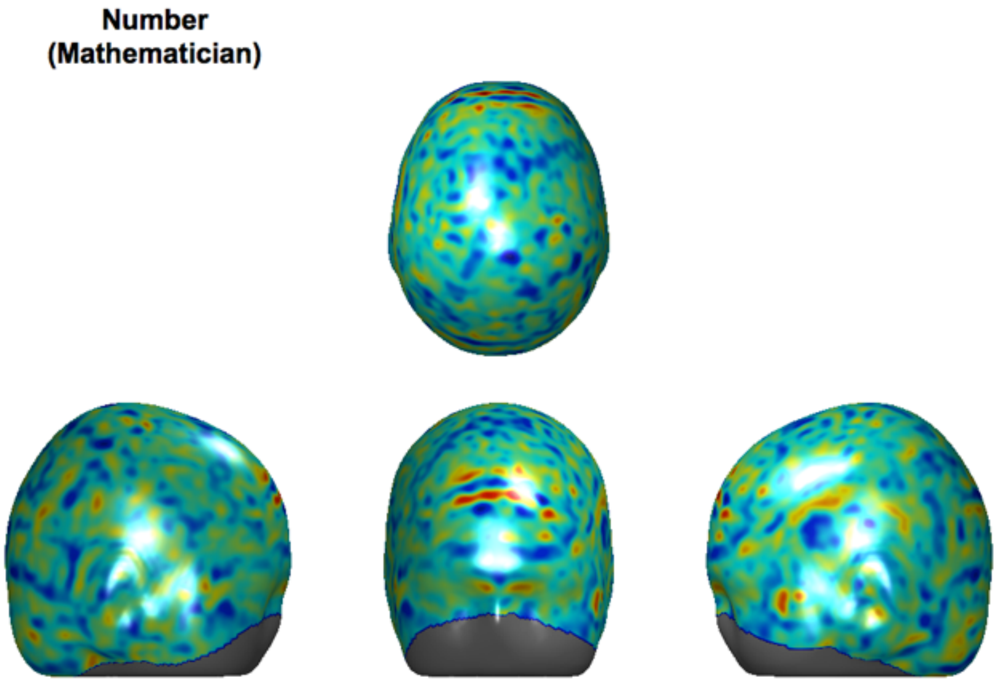

Supplementary Figure 18: Faculty XVIII.

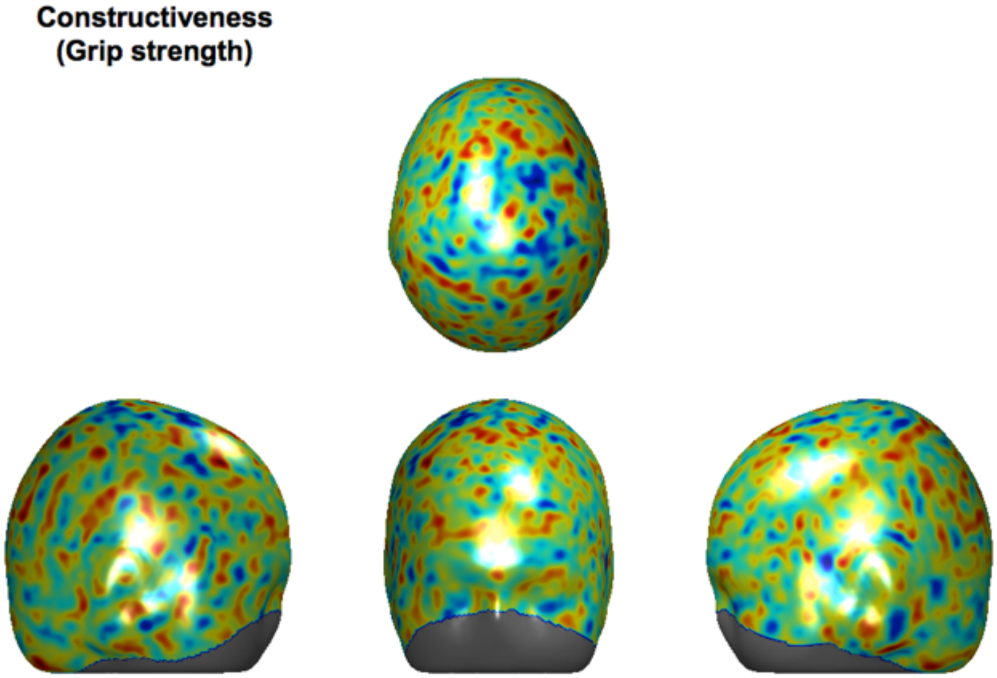

Supplementary Figure 19: Faculty XIX.

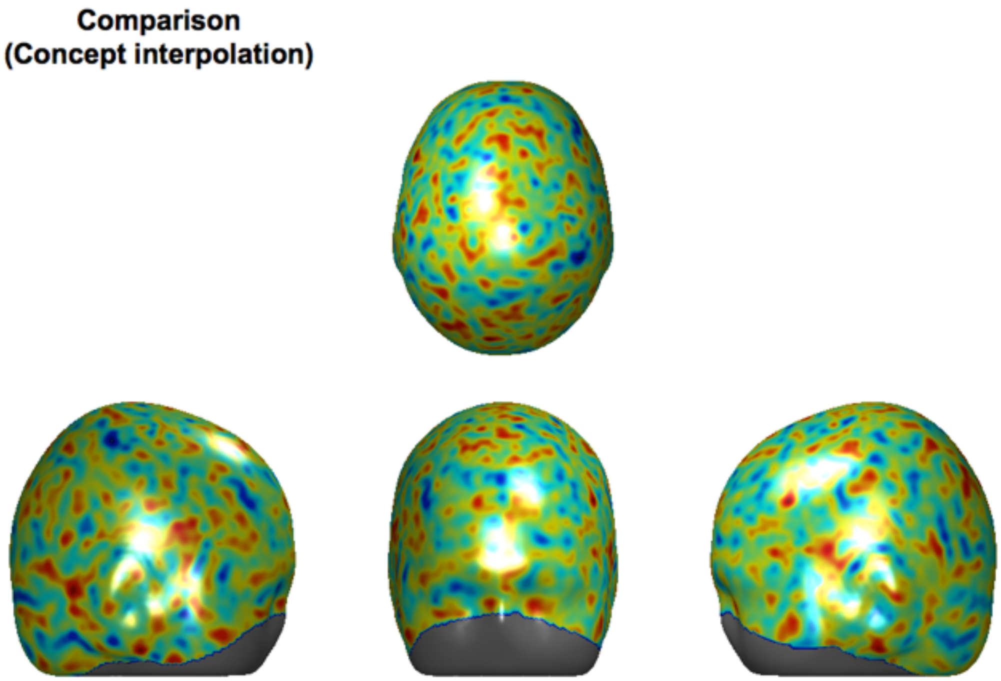

Supplementary Figure 20: Faculty XX.

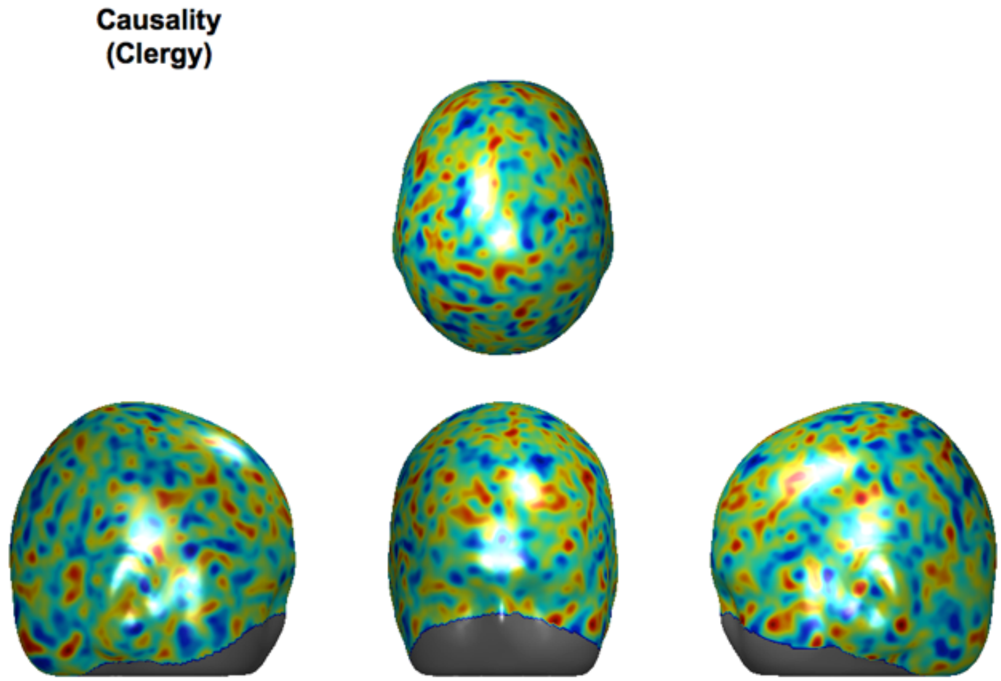

Supplementary Figure 21: Faculty XXI.

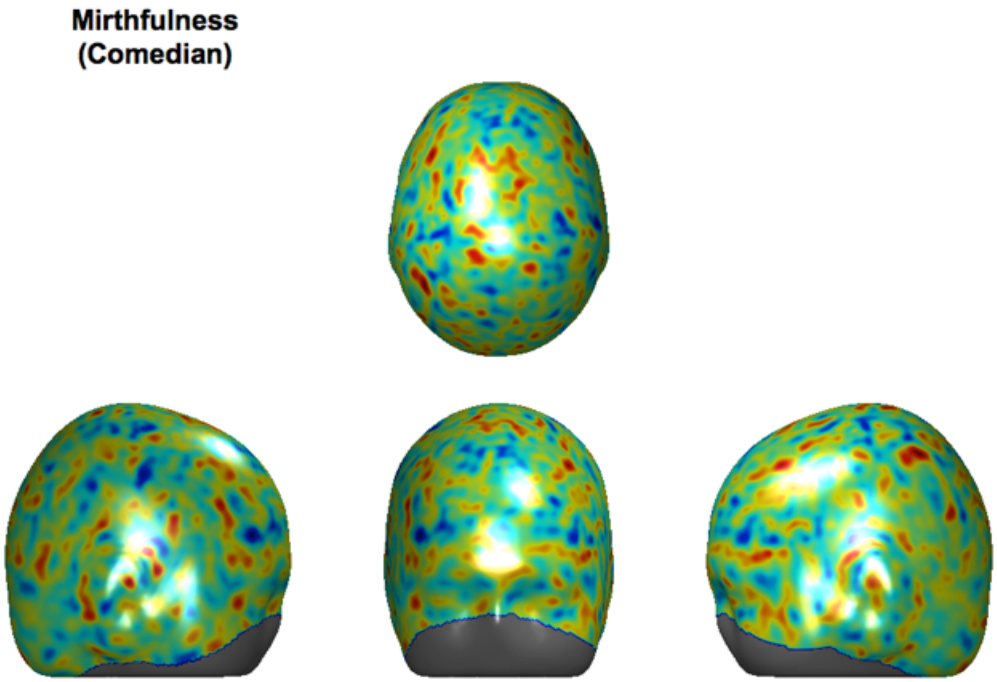

Supplementary Figure 22: Faculty XXII.

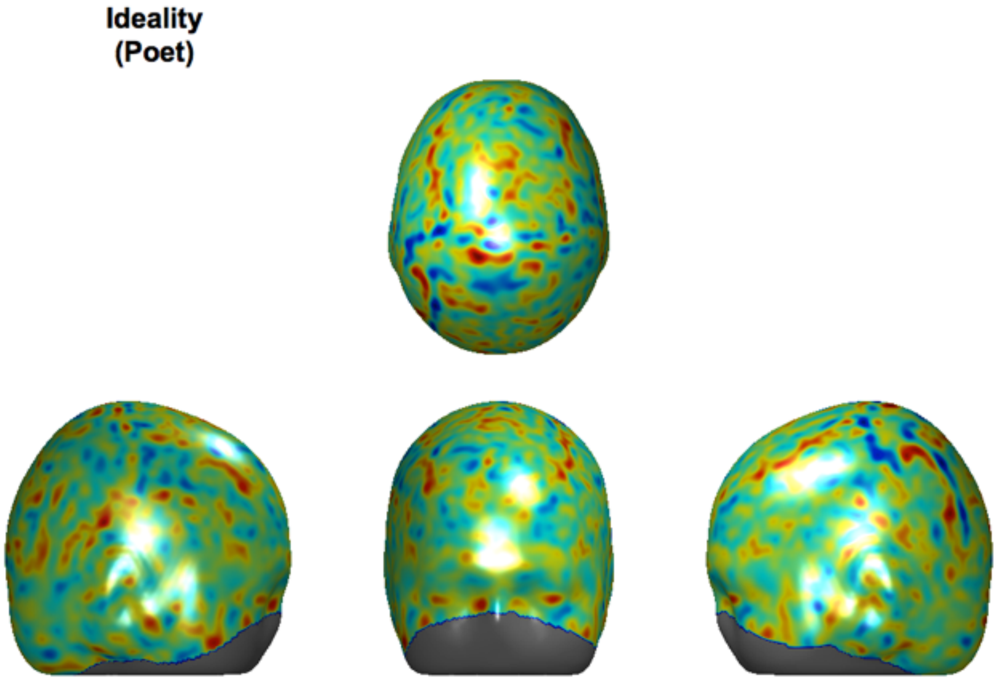

Supplementary Figure 23: Faculty XXIII.

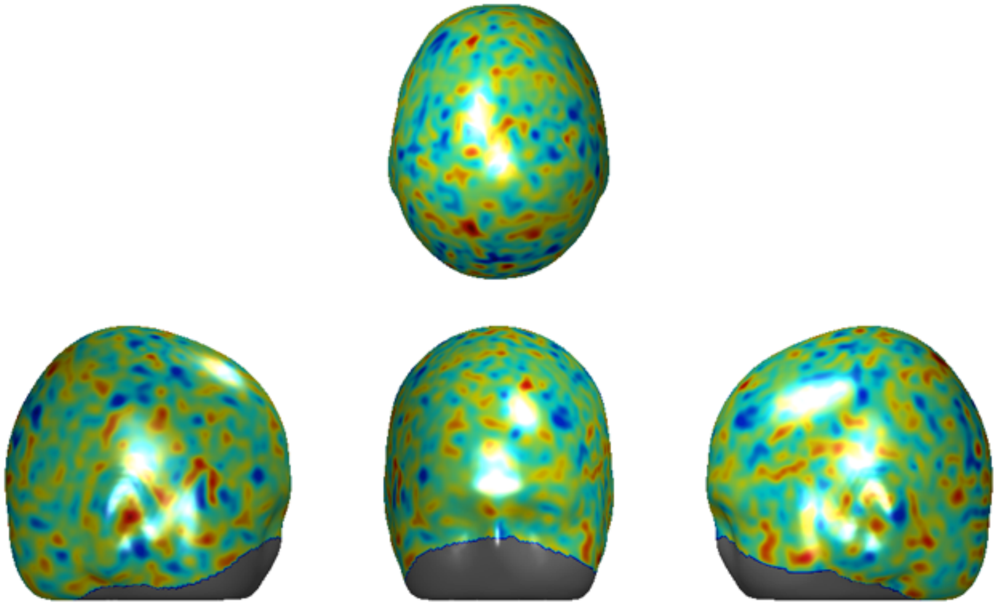

Supplementary Figure 24: Faculty XXIV.

Supplementary References

Gall, F. J. (1835). *On the functions of the brain and each of its parts: With observations on the possibility of determining the instincts, propensities, and talents, or the moral and intellectual dispositions of men and animals, by the configuration of the brain and head*. (W. Lewis, Ed.). Boston, MA: Marsh, Capen and Lyon.

Spurzheim, J. G. (1815). *The physiognomical system of Drs. Gall and Spurzheim; founded on an anatomical and physiological examination of the nervous system in general, and of the brain in particular; and indicating the dispositions and manifestations of the mind*. London: Baldwin, Cradock, and Joy.

Wilkes, J. (1825). *Encyclopaedia Londinensis, Or, Universal Dictionary of Arts, Sciences, and Literature* (Volume XX). London: J. and C. Adlard.
